# Precision Rehabilitation in Spinal Cord Injury: A Systematic Review of Omics Applications for Intervention Monitoring in Spinal Cord Injury

**DOI:** 10.1016/j.arrct.2026.100598

**Published:** 2026-02-16

**Authors:** Alexander Stacul, Ezra Valido, Nicole Nyfeler, Alessandro Bertolo, Ramona M. Zeh, Andrea O. Fontana, Jürgen Pannek, Jörg Krebs, Alexander Leichtle, Marija Glisic, Jivko Stoyanov

**Affiliations:** a SCI Population Biobanking and Translational Research, Swiss Paraplegic Research, Nottwil, Switzerland; b Graduate School for Cellular and Biomedical Sciences, University of Bern, Bern, Switzerland; c Institute of Social and Preventive Medicine, University of Bern, Bern, Switzerland; d Swiss Paraplegic Center, Nottwil, Switzerland; e Department of Neuro-Urology, Swiss Paraplegic Center, Nottwil, Switzerland; f Neuro-Urology, Swiss Paraplegic Research, Nottwil, Switzerland; g Department of Urology, Inselspital, Bern University Hospital, University of Bern, Bern, Switzerland; h Zentrallabor, Cantonal Hospital Baden (KSB), Baden, Switzerland; i Institute for Laboratory Medicine and Microbiology, University Hospital Augsburg, Augsburg, Germany; j Multidisciplinary Center for Infectious Diseases, University of Bern, Bern, Switzerland; k Cardiometabolic and Respiratory Research, Swiss Paraplegic Research, Nottwil, Switzerland

**Keywords:** Intervention, Omics, Rehabilitation, Rehabilitation outcome, Spinal cord injury

## Abstract

**Objective:**

To systematically evaluate the application and utility of omics technologies, high-throughput methods measuring the complete or targeted set of molecules inside a biological system at a certain timepoint, in monitoring and optimizing rehabilitation interventions in traumatic spinal cord injury.

**Data sources:**

Embase, Medline/Ovid, and Web of Science were searched from inception to November 27, 2024.

**Study Selection:**

Eligible studies included adults (≥18 years) with spinal cord injury undergoing rehabilitation interventions assessed using omics technologies (genomics, epigenomics, transcriptomics, proteomics, metabolomics, or metagenomics).

**Data Extraction:**

Following PRISMA guidelines, independent screening, data extraction, and risk of bias (RoB) assessment (National Institutes of Health Quality Assessment Tools) were performed by 2 investigators. Based on RoB assessment, studies were classified from level 1 (most reliable) to level 4 (least reliable).

**Data synthesis:**

Twenty-three trials were included: 8 randomized controlled trials, 5 non-randomized controlled trials, and 10 pre-post trials. Twenty-two studies (96%) exhibit a moderate RoB due to small sample size and heterogeneity. Omics technologies were primarily applied to exercise and electrical muscle stimulation interventions (65%), followed by hormonal and cellular therapies (22%), and diet (13%). Transcriptomic analyses revealed consistent molecular adaptations, including increased mitochondrial biogenesis (proliferator-activated receptor gamma coactivator 1-alpha) and reduced muscle atrophy gene expression (myostatin), correlating with enhanced insulin sensitivity and improved aerobic capacity. Metagenomics consistently identified microbiome shifts, such as decreased inflammatory taxa and increased beneficial taxa, associated with improved metabolic profiles and bowel function. Proteomics and metabolomics highlighted systemic changes related to neurorecovery, immune modulation, and sperm motility, linking molecular signatures directly to clinical outcomes.

**Conclusions:**

Omics technologies enable early identification of molecular alterations. However, given small sample sizes and heterogeneity of the current studies, these findings should be interpreted with caution. Gradual integration of omics, particularly epigenomics which may capture long-term, injury-related changes holds promise for developing personalized rehabilitation protocols and monitoring clinical progression in spinal cord injury.

Spinal cord injury (SCI) is a severe condition that can lead to temporal or permanent disruption of sensory, motor, and autonomic functions, impairing the quality of life of patients and challenging the efficacy of rehabilitation strategies.^1^ Although advancements in neurorehabilitation have progressively improved patient outcomes, functional recovery remains substantially limited due to the complex and heterogenous biological processes involved in neuronal regeneration, neuroplasticity, and systemic adaptation after injury.^2^^,^^3^ Rehabilitation interventions typically focus on enhancing motor function, managing secondary complications such as cardiovascular, urinary and gastrointestinal dysfunction, and improving overall patient independence.^4^ Nevertheless, the clinical effectiveness often varies markedly, influenced by the interactions between injury-specific factors and the individual’s unique biological responses. Conventional clinical outcome measures, predominantly relying on physical and functional assessments, frequently fail to detect subtle yet potentially critical molecular and physiological changes occurring early in the rehabilitation process. This limitation restricts the capability of clinicians to precisely tailor interventions and monitor their efficacy at the biological level, potentially overlooking early indicators of recovery or deterioration.^5^^,^^6^

Emerging evidence highlights the importance of integrating molecular profiling and biomarker analysis into rehabilitation research, providing a detailed understanding of the biological foundations influencing the response to therapeutic interventions. Omics technologies, referring to the large-scale, comprehensive study of a complete or targeted set of molecules inside a biological system at a certain timepoint, comprises the study of an organisms entire genome (genomics),^7^ the investigation of genome-wide epigenetic modifications (epigenomics),^7^ the analysis of the genomes within microbial communities (metagenomics),^8^ the comprehensive examination of the RNA transcript (transcriptomics),^7^ the large-scale or targeted study of the protein complement (proteomics),^7^ and the analysis of metabolites (metabolomics),^7^ have revolutionized biomedical research, offering comprehensive insights into molecular processes associated with neurologic injuries and recovery trajectories.^9^ By systematically characterizing biological molecules, these technologies facilitate the identification of molecular pathways involved in SCI pathology, including neuroinflammation,^10^^,^^11^ cellular repair and regeneration mechanisms,^12^ and metabolic changes.^13^ The application of omics approaches thereby enables researchers and clinicians to identify biomarkers predictive of rehabilitation outcomes, ultimately informing the optimization of therapeutic strategies through personalized intervention protocols.

In other areas of neurorehabilitation, notably stroke and traumatic brain injury, omics-based methodologies have successfully demonstrated their potential in enhancing the precision and individualization of rehabilitation practices. For example, transcriptomic profiling in stroke rehabilitation has elucidated pathways associated with neuroplasticity and motor function restoration, enabling precise targeting of therapeutic interventions and individualized patient management strategies.^7^ Similarly, microbiome analysis in traumatic brain injury rehabilitation has uncovered interactions between gut microbiota and systemic inflammation, providing targets for dietary or microbial interventions aimed at reducing inflammatory responses and improving functional recovery.^8^ Such successful integration in related neurologic fields highlights the translational potential of omics technologies within SCI rehabilitation. However, for the SCI population due to systemic challenges (high biological heterogeneity: eg, lesion level, time since injury; lack of unified protocols: eg, biospecimen collection, bioinformatics processing) these technologies are not yet used in a standardized manner.

The present systematic review aims to critically evaluate and summarize the current application of various omics platforms in monitoring and optimizing rehabilitation interventions in individuals with traumatic SCI. Furthermore, this review assesses the efficacy of diverse intervention types, specifically, exercise and electrical muscle stimulation, dietary modification, and pharmacologic, hormonal, and cellular therapies, by examining molecular adaptations identified through omics analyses. Such a synthesis is essential to identify molecular markers linked with improved clinical outcomes, thereby facilitating their translation into clinical rehabilitation practice and contributing to the advancement of precision medicine in SCI management.

## Methods

### Data sources and search strategy

This systematic review was conducted according to the Preferred Reporting Items for Systematic Reviews and Meta-Analyses (PRISMA) guidelines,^14^ and adhered to the recommendations provided by the “24-step guide for conducting systematic reviews and meta-analyses in medical research.”^15^ A comprehensive literature search was performed across 3 electronic databases: EMBASE (Elsevier), MEDLINE (National Library of Medicine) and Web of Science (Thomson Reuters)—from inception until November 27, 2024, without language restrictions. To ensure thorough coverage, the reference lists of eligible articles were reviewed for potential additional studies. The complete search strategy, including specific terms related to omics technologies (genomics, epigenomics, metagenomics, transcriptomics, proteomics, and metabolomics), rehabilitation interventions, and SCI, is detailed in supplemental appendix S1 (available online only at http://www.archives-pmr.org/).

### Study selection, eligibility criteria and data extraction

The review protocol was registered prospectively with PROSPERO (ID CRD42023404178). Eligible studies for inclusion were required to: (i) recruit adult participants (≥18 years old) with traumatic SCI; (ii) employ an intervention (randomized or non-randomized clinical trials with or without control group) that targeted rehabilitation outcomes; (iii) use one or more omics methodologies (genomics, epigenomics, metagenomics, transcriptomics, proteomics, or metabolomics) to assess intervention-related molecular outcomes; and (iv) were published in peer-reviewed journals.

We excluded studies if they involved animal models, were published as letters to the editor, reviews, commentaries, and conference abstracts or lacked clearly defined interventions. Titles and abstracts were independently screened by 2 reviewers to determine initial eligibility. Relevant articles subsequently underwent full-text assessment by the same reviewers, who verified adherence to the predefined inclusion and exclusion criteria. Disagreements at either stage were resolved by discussion until consensus was reached, with arbitration from a third reviewer where necessary.

Data extraction was performed independently by 2 reviewers using a structured data extraction form, capturing the following details: lead author and publication year, study design, country of origin, sample size, participant characteristics (age, sex, duration and completeness of injury), specific rehabilitation intervention employed, omics methodologies used, biological samples analyzed, rehabilitation outcomes evaluated, and principal molecular findings. Any discrepancies arising during data extraction were resolved by consensus or consultation with a third reviewer.

### Methodological quality assessment

To assess the methodological rigor of included studies, quality appraisal was conducted using standardized tools developed by the National Institutes of Health (NIH). The NIH Quality Assessment Tools were selected because they provide structured, domain-specific appraisal criteria for RCTs, non-RCTs, and pre-post designs, which reflect the methodological diversity inherent to rehabilitation research. NIH tools were used to classify risk of bias (RoB), not to infer their validity specifically for omics methodologies. For studies with controlled intervention designs (RCTs and non-RCTS), the NIH Quality Assessment Tool for Controlled Intervention Studies was employed, which focuses on key aspects such as randomization, blinding, and outcome reporting.^16^ In cases where studies lacked a control group, the NIH Quality Assessment Tool for Before-After (Pre-Post) Studies with No Control Group was used.^17^ This tool assesses elements such as the clarity of the study question, eligibility criteria, and consistency in outcome measurements before and after the intervention.

Two reviewers independently assessed the quality of the studies and their classification simultaneously, and any disagreements or discrepancies were resolved by reaching a consensus or by consulting a third reviewer. Quality ratings were categorized as low, moderate, or high RoB. Later (regarding RoB, strength, and study design), classification of studies going from level 1 to level 4 was done. Level 1 studies indicating the most reliable studies, and level 4 least reliable studies. Detailed quality assessment findings and classifications are reported systematically for each study within the results and supplementary materials (supplemental tables S1 and S2, available online only at http://www.archives-pmr.org/).

### Synthesis of evidence

Because of the heterogeneity in study population, trial design, and reported health outcomes, statistical meta-analysis was not appropriate. Instead, we used a narrative synthesis of the evidence to give a picture of current omics platforms and their efficacy in monitoring diverse intervention types. We created an evidence summary highlighting both the potential and methodological quality of the underlying studies. Findings from the highest-quality interventional studies (level 1-2) are presented in the main text, as these trials (particularly randomized controlled trials) carry a lower RoB and offer the most robust evidence. Evidence from non-RCTs and pre-post designs are discussed more briefly. These studies can also be positioned as preliminary indications that require confirmation in future RCTs.

## Results

### Literature search and study characteristics

**Fig 1:**
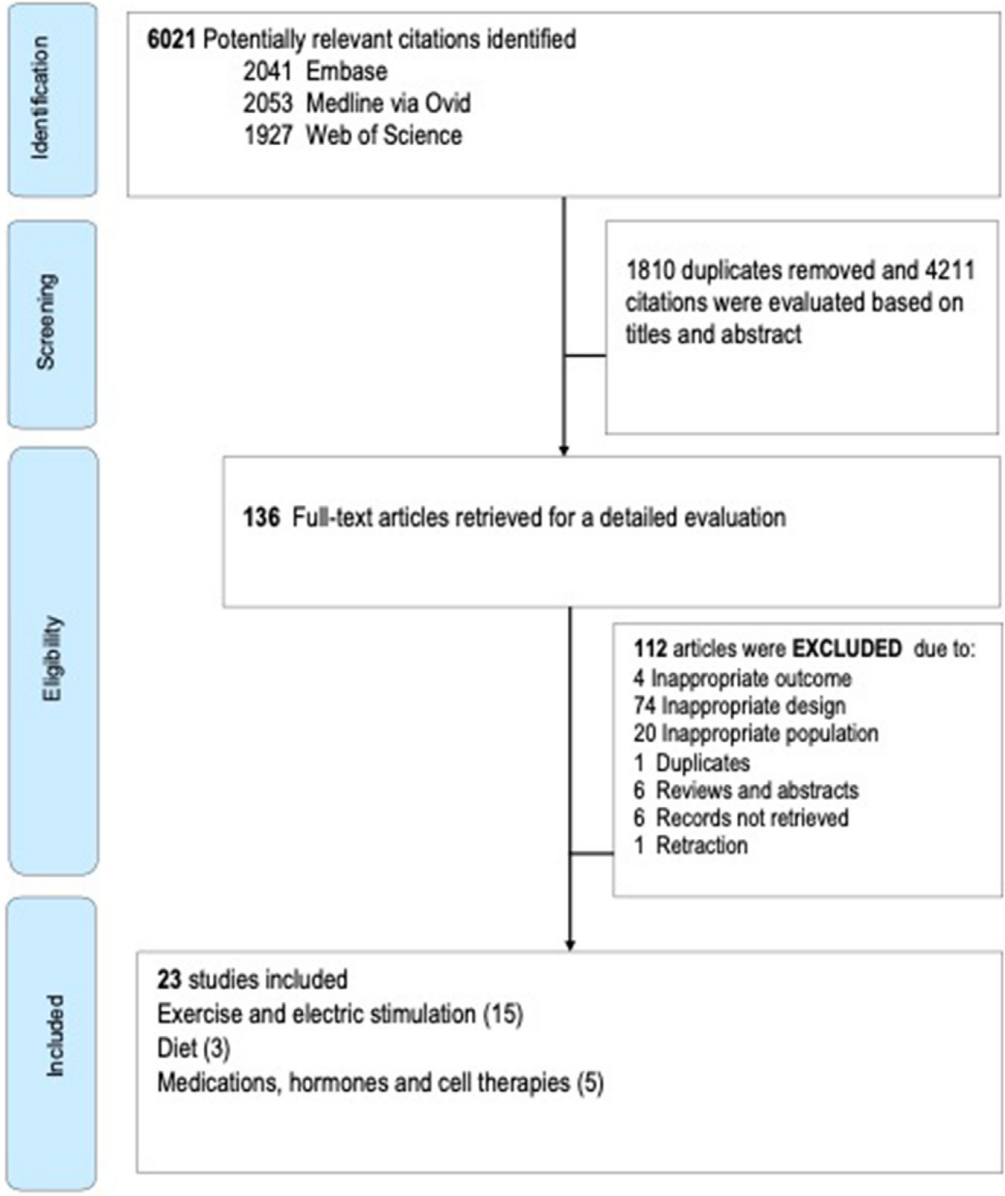
Flowchart of search strategy. From EMBASE, MEDLINE/Ovid and Web of Science, 6021 citations were identified, 4211 citations were evaluated based on title and abstract, 136 full-text articles assessed, and a total of 23 studies were included in this review.

The initial search retrieved 6021 references (figure 1). After removing 1810 duplicates, 4211 titles and abstracts were screened. Of these, 136 full-text articles were assessed in detail, resulting in the final inclusion of 23 trials meeting the defined eligibility criteria. Exclusion of 112 articles was due to inappropriate outcome, design, and population, retraction, records not retrieved, reviews and abstracts, and duplicates. Reference lists of the selected studies were reviewed, but did not yield additional eligible articles.

Study characteristics, participants, and summary of the included trials included in this review are shown in tables 1-3.^18^, ^19^, ^20^, ^21^, ^22^, ^23^, ^24^, ^25^, ^26^, ^27^, ^28^, ^29^, ^30^, ^31^, ^32^, ^33^, ^34^, ^35^, ^36^, ^37^, ^38^, ^39^, ^40^ The included studies comprised 8 RCTs (35%), 5 non-RCTs (22%), and 10 pre-post intervention studies without control groups (43%). Most excluded articles (66%) were disqualified due to unsuitable study designs, notably retrospective analyses, or the absence of clearly defined interventions. Of the included trials, 15 (65%) involved exercise and electrical muscle stimulation interventions, 3 (13%) examined dietary interventions, and 5 (22%) focused on medications, hormones, or cellular therapies.

**Table 1:** Summary of the findings for the exercise and electrical muscle stimulation intervention trials included in the review

Author, Publication Year, Country, Type of Study	SCI Population, Age (SD)/(Range), Male (%)	Intervention	Sample, Organ-System, Omics Measured	Significant Findings (↑) Upregulation, (↓) Downregulation	RoB Level
Hu et al,^18^ 2024, China, RCT	20 (complete SCI), NA, NA	Exoskeleton training	Stool, gastrointestinal, metagenomics	(↑) *Firmicutes, Proteobacteria, Actinobacteria Ruminococcus 1, Ruminococcaceae UCG002, Faecalibacterium, Dialister, Ralstonia, Escherichia-Shigella, Bifidobacterium* (↓) *Bacteroidetes, Verrucomicrobia, Bacteroides, Prevotella, Parabacteroides, Akkermansia, Blautia, Ruminococcus 2, Megamonas*	Moderate 2
Nightingale et al,^19^ 2017, United Kingdom, RCT	21 (CON: 8, INT: 13) (chronic SCI), CON: 48 (10), INT: 46 (6), 15 (71.4) CON: 6 (75), INT: 9 (69.2)	Home-based moderate-intensity upper-body exercise (INT) or a lifestyle maintenance (CON)	Blood and adipose tissue, cardiovascular and metabolic, transcriptomics	(↓) lipid metabolism regulator (adipose tissue triglyceride lipase)	Moderate 2
Goldhardt et al,^20^ 2019, Brazil, RCT	10, 40.6 (9.03), 5 (50)	Gait training	Peripheral blood, neurologic and cardiovascular, epigenomics	TS: (↑) oxidative stress markers (AOPP, nitrite, TBARS) WS: (↑) oxidative stress markers (AOPP, nitrite), antioxidant markers (GSH, CAT)	Moderate 2
Petrie et al,^21^ 2014, United States, non-RCT	9, 36 (19-63), 9 (100)	Electrical induced exercise	Muscle tissue, musculoskeletal, transcriptomics	Before exposure: (↑) muscle atrophy markers (MSTN, ANKRD1), glycolytic muscle properties (MYH8, MYCBP2) (↓) oxidative processes and slow-twitch properties (MYL3, SDHB, PDK2 and RyR1) After exposure: (↓) muscle atrophy markers (MSTN, ANKRD1), glycolytic muscle properties (MYH8, MYCBP2)	Moderate 3
Petrie et al,^22^ 2014, United States, pre-post study	5 (complete paraplegia), 30.4 (4.39), NR	Electrical induced exercise	Muscle tissue, musculoskeletal, transcriptomics	Acute stimulation: (↑) oxidative regulators (PGC-1α, NR4A3, IFRD1, ABRA, PDK4) (↓) muscle atrophy markers (MSTN) Chronic stimulation: (↑) expression related in glycolysis (PDHA1, PDHB, PDHX), fatty acid oxidation (ACADVL, ACAD8, ACAD9), tricarboxylic acid (BRP44, BRP44L, OGDH, SDHB), oxidative genes (NDUFB1, NDUFA2, CYC1, COQ10A), oxidative muscle fiber (MYH6, MYH7 MYL3, MYL2), mitochondrial fission/fusion (MFF, OA1, MFN1, MFN2) (↓) glycolytic muscle fibers (ACTN3, MYLK2, MYL5), muscle atrophy marker (MSTN)	Moderate 4
Petrie et al,^23^ 2015, United States, pre-post study	12 (chronic complete SCI)), NR, NR	Electrical induced exercise	Muscle tissue, musculoskeletal, transcriptomics	(↑) oxidative regulators (PGC-1α, NR4A3, ABRA)	Moderate 4
Petrie et al,^24^ 2016, United States, non-RCT	5 (chronic SCI), 30.6 (24-39), 5 (100)	Vibration stimulation or electrical induced exercise or heat stress	Muscle tissue, musculoskeletal, transcriptomics	Electrical induced exercise: (↑) oxidative regulators (NR4A3, PGC-1α, ABRA) Vibration: (↑) muscle metabolism regulator (FOXK2) Electrical induced exercise and vibration: (↓) muscle atrophy marker (MSTN) Heat stress: (↓) oxidative metabolism regulator (PGC-1α), muscle atrophy marker (ANKRD1)	Moderate 3
Petrie et al,^25^ 2020, United States, pre-post study	15 (8 ST, 7 LT), 36 (12) (ST: 37.9 (16.1), LT: 32.3 (6.8)), 15 (100)	Electrical induced exercise	Muscle tissue, musculoskeletal, transcriptomics and epigenomics	(↑) mitochondrial biogenesis and metabolism (PGC-1α, PRKAB2), muscle small molecule transport (FNDC5) (↓) muscle atrophy marker (MSTN)	Moderate 4
Petrie et al,^26^ 2022 United States, Pre-post study	17 (1Hz: 9, 3Hz: 10, 5 Hz: 7, 20Hz: 12), (1Hz: 39.5 (8.5), 3Hz: 38.9 (8.2), 5Hz: 34.9 (13.7), 20Hz: 32.6 (9.8)), NR	Electrical induced exercise	Muscle tissue, musculoskeletal, transcriptomics and epigenomics	(↑) oxidative metabolism regulator (PGC-1α) (↓) muscle atrophy marker (MSTN)	Moderate 4
Petrie et al,^27^ 2024, United States, pre-post study	10 (1Hz: 9, 3Hz: 10) (complete SCI), 1Hz: 39.5 (8), 3Hz: 38.8 (7.8), 10 (100)	Electrical induced exercise	Muscle tissue, musculoskeletal, transcriptomics and epigenomics	3Hz: (↑) mitochondrial biogenesis and metabolism (NR4A3, PGC-1α, ABRA, IRS2, EGR1) (↓) muscle atrophy marker (MSTN)	Moderate 4
Hjeltnes et al,^28^ 1999, Sweden, non-RCT	7 (tetraplegic), 35 (2), 7 (100)	Electrically stimulated leg cycling training	Muscle biopsy, musculoskeletal, transcriptomics	(↓) energy metabolism regulators (UCP2, UCP3)	Moderate 3
Lammers et al,^29^ 2012, Netherlands, non-RCT	8, 39 (3), NR	Electrical induced exercise	Muscle tissue, metabolic and musculoskeletal, transcriptomics	Recovery: (↑) fatty acid metabolism (FAB3, HADH, SLC25A20) Deconditioning: (↓) fatty acid metabolism (FAB3, HADH, SLC25A20)	Moderate 3
Vissing et al,^30^ 2005, Denmark, Pre-post study	6, 39 (9), 6 (100)	Electrical induced exercise	Muscle biopsy, musculoskeletal, transcriptomics	(↑) myogenesis and oxidative metabolism (Myogenin, MHC I, HADHA, CS, mFABP) (↓) fast muscle fiber and glycolysis markers (MHC IIx, GOPDH)	Moderate 4
Ingles et al,^31^ 2016, Spain, pre-post study	16 (paraplegic using wheelchairs for mobility), 42.1 (27-68), 14 (87.5)	Physical exercise (Graded exercise test)	Blood and capillary blood lactate, neuromuscular and metabolic, transcriptomics	Active group: (↑) antioxidant response (Catalase, GPx), oxidative damage markers (MDA, protein carbonyls)	Moderate 4
Chang et al,^32^ 2012, United States, pre-post study	5 (chronic complete SCI), 31.1 (8.2) (21-54), NA	Vibratory stimulation	Muscle biopsy, neuromuscular, transcriptomics	(↑) neuronal plasticity (ERK1) (↓) neurotransmission regulators (GLRA3, GRM7, SYN3, CDH2)	Moderate 4

*Abbreviations:* AOPP = advanced oxidation protein products; ASCI = acute spinal cord injury; CAT = catalase; CON = control group; FES = functional electrical stimulation; GPx = glutathione peroxidase; GSH = reduced glutathione; INT = intervention group; LT = long-term; MDA = malondialdehyde; NA = not applicable; NR = not reported; RCT = randomized controlled trial; RoB = risk of bias; SCI = spinal cord injury; ST = short-term; TBARS = thiobarbituric acid reactive substances.

Five omics methodologies were reported across the studies: transcriptomics (11 trials, 48%), metagenomics (4 trials, 17%), metabolomics (2 trials, 9%), proteomics (2 trials, 9%), and epigenomics (1 trial, 4%). Three trials (13%) employed a combined approach using both transcriptomics and epigenomics.

### Methodological quality assessment of included studies: RoB

Methodological quality was independently assessed by 2 reviewers using the NIH Quality Assessment Tools corresponding to each study design. No discrepancies arose and had to be resolved through consultation with a third reviewer. Formal inter-rater reliability statistics were not calculated, as final ratings were based on consensus judgments, in line with common practice for reviews using the NIH tools. Based on the quality assessment from the NIH, among the interventional clinical trials (pre-post, randomized, non-randomized), including 8 RCTs, 5 non-RCTS and 10 pre-post studies without a control group, 96% (*n* = 22) had a moderate RoB. Only one RCT was rated as low RoB. For the controlled clinical trials (RCTs and non-RCTs), the major issues driving this assessment comprised incomplete reporting of randomization methods, allocation concealment, and blinding procedures. Major issues relating to the pre-post trials without a control group were commonly due to inadequate justification of sample size, individual-level data, intervention consistency, and blinding. Classification of the studies from level 1 to level 4 was according to study design and RoB assessment. Among controlled clinical trials, 1 RCT was classified as level 1, the other 7 as level 2, and all the non-RCTs as level 3. All pre-post trials without a control group were classified as level 4 (tables 1-3; supplemental tables S1 and S2, available online only at http://www.archives-pmr.org/).

### Exercise and electrical muscle stimulation

Fifteen trials (65%) examined exercise-based and electrical stimulation interventions, including functional electrical stimulation (FES), exoskeleton-assisted walking, aerobic exercise, whole-body vibration, and isolated muscle contractions (table 1). Among these, 3 were RCTs, 4 were non-RCTs, and 8 were pre-post intervention studies without controls. Omics methods used in these studies included transcriptomics, metagenomics, and epigenomics, with a focus on evaluating muscular, gastrointestinal, and systemic biological changes. Clinical outcomes assessed included changes in bowel function, insulin sensitivity, cardiorespiratory fitness, and oxidative stress profiling.

In a metagenomics-based RCT by Hu et al,^18^ exoskeleton-assisted walking induced changes in gut microbiota composition among individuals with complete SCI, mainly with an increase in beneficial taxa. Clinically, this correlated with improved bowel function (Neurogenic Bowel Dysfunction score), including reduced defecation time and improved bowel frequency.

Using transcriptomics, Nightingale et al,^19^ demonstrated that moderate-intensity upper-body exercise in chronic SCI significantly reduced adipose tissue triglyceride lipase expression, correlating with enhanced insulin sensitivity (HOMA2-IR) and cardiorespiratory fitness (VO_2_-max peak).

An epigenomics approach by Goldhardt et al,^20^ identified differential oxidative stress responses after a single treadmill or walker-assisted training session, highlighting molecular differences in exercise modalities. No significant changes were found in brain-derived neurotrophic factor levels and histone acetylation. In contrast, walker-based training was associated with upregulation of antioxidant defense.

The 4 non-RCTs and 8 pre-post trials without a control arm used either transcriptomics as a single approach or in combination with epigenomics. They reported on metabolic regulation and mitochondrial adaptation, oxidative stress, and neuromuscular plasticity.

Seven related studies by Petrie et al,^21^, ^22^, ^23^, ^24^, ^25^, ^26^, ^27^ used transcriptomics and epigenomics to examine the molecular effects of various electrical stimulation protocols on paralyzed muscle. Repeated electrical stimulation consistently upregulated mitochondrial biogenesis markers, particularly peroxisome proliferator-activated receptor gamma coactivator 1-alpha (PGC-1α), and downregulated muscle atrophy-related genes such as myostatin (MSTN). Chronic stimulation produced epigenetic modifications, such as DNA promoter demethylation, suggesting long-term adaptive changes and transcriptional reprogramming toward oxidative metabolism pathways. Acute or chronic stimulation led to distinct gene expression signatures. Fatiguing contractions preferentially activated stress pathways, whereas non-fatiguing protocols enhanced metabolic and contractile gene expression. Comparisons between vibration, heat stress, and electrical stimulation revealed contraction mode-specific gene expression, suggesting selective pathways related to input type.

In later studies, epigenomic analysis showed that chronic stimulation over weeks led to demethylation of gene promoters (mainly PGC-1α), indicating transcriptional reprogramming, which aligns with upregulation of oxidative metabolism genes. Specifically, in 1 trial, compared to the control group, 74 pathways were significantly demethylated and were associated with signal transduction, gene expression, and transcription of the immune system and metabolism. Acute stimulation, despite gene expression changes, did not produce measurable changes, highlighting a dose-dependent threshold for epigenetic remodeling. Their most recent work compared 1 Hz versus 3 Hz low-force isometric stimulation, reporting that 3 Hz for 1 hour elicited stronger acute expression of early response genes involved in glucose signaling, mitochondrial regulation, contractile function, and muscle remodeling. These findings demonstrate that even small frequency adjustments can amplify beneficial molecular responses.

Hjeltnes et al^28^ showed that FES-cycling in individuals with tetraplegia leads to a reduction in uncoupling protein 2 (UCP2) and uncoupling protein 3 (UCP3) mRNA expression (mitochondrial proteins associated with oxidation). Also, whole-body insulin stimulated glucose uptake and expression of genes involved in glucose metabolism was upregulated.

Lammers et al^29^ demonstrated that FES restored expression of metabolic genes in individuals with SCI, reversing the downregulation caused by disuse. Transcriptomic profiling revealed that disuse downregulated genes involved in fatty acid oxidation and insulin signaling, while FES restored or enhanced their expression, suggesting beneficial effects of FES on metabolic health.

Vissing et al^30^ applied low-frequency FES in males with SCI. Over the intervention period, they observed a significant increase in a key myogenic regulator and a shift toward an oxidative muscle phenotype, with early tendencies for other oxidative markers to increase and fast fiber-specific markers to decrease after 2 weeks.

Using the Borg-Rating of Perceived Exertion scale, Ingles et al^31^ monitored individuals with paraplegia (using wheelchairs for mobility), undergoing a graded exercise test until volitional exhaustion. Plasma markers of oxidative damage significantly increased immediately post-exercise. Active paraplegic individuals exhibited lower exercise-induced oxidative damage and a higher expression of antioxidants.

Finally, Chang et al^32^ investigated neuromuscular responses during limb segment vibration. They observed a significant depression of the H-reflex during vibration in both groups, with the control group demonstrating longer-lasting inhibition. Furthermore, the vibration intervention was associated with significant alterations in neuromuscular markers, with 4 decreasing (GLRA3, GRM7, SYN3, and CDH2) and 1 (ERK1) increasing.

### Dietary interventions

Three RCTs (13%) assessed dietary interventions employing metagenomics and metabolomics, focusing on microbiome composition, inflammation, metabolic function, and motor recovery. Clinical outcomes assessed included bowel function, inflammatory status, insulin sensitivity, and motor recovery (table 2).

**Table 2:** Summary of the findings for the dietary intervention studies included in the systematic review

Author, Publication Year, Country, Type of Study	SCI Population, Age (SD)/(Range), Male (%)	Intervention	Sample, Organ-System, Omics Measured	Significant Findings (↑) Upregulation, (↓) Downregulation	RoB Level
Li et al,^33^ 2022, United States, RCT	19 (INT: 8, CON: 11) (chronic SCI), INT: 44.8 (11.1), CON: 41.1 (13.2), 13 (68.4) (INT: 6 (75), CON: 7 (63.6))	Low-carbohydrate, high-protein Diet	Stool, gastrointestinal, metagenomics	(↑) *Bacteroides thetaiotaomicron, Coprococcus 3, Fusicatenibacter* (↓) *Tyzzerella, Phascolarctobacterium, Clostridium sensu stricto 1*	Low 1
Valido et al,^34^ 2024, Switzerland, pilot RCT	11 (traumatic SCI), NA, NA	Probiotic vs prebiotic	Stool, gastrointestinal, metagenomics	Probiotic: (↑)* 5/30 inflammatory markers: growth factors (Angiopoietin2, SCF, TGFα, GM-CSF), metabolic factors (Adipsin) (↓)* 25/30 inflammatory markers: essential immune response (IL1β, IL2, IL4, IL6, IL8, IL10, IL12p70, IL17A, IFNγ, IP10, MCP1, TNFα, TGFβ1, CRP), growth factors (PDGF-AA, PDGF-BB, VEGF, HGF, EGF, EPO, FGF-basic, G-CSF, M-CSF), metabolic factors (Leptin, Resistin)	Moderate 2
Yarar-Fisher et al,^35^ 2018 United States, RCT	7 (incomplete SCI), 35 (12), 5 (71.4)	KD vs standard diet	Blood, inflammatory, metabolomics	(↑) anti-inflammatory lysophospholipid (LysoPC 16:0) (↓) anti-inflammatory blood protein (Fibrinogen)	Moderate 2

*Abbreviations:* CON = control group; INT = intervention group; KD = ketogenic diet; NA = not applicable; RCT = randomized controlled trial; RoB = risk of bias; SCI = spinal cord injury.

⁎ Not statistically significant.

Li et al^33^ examined a low-carbohydrate, high-protein diet in chronic SCI, demonstrating significant reductions in pro-inflammatory gut bacteria and enhanced insulin sensitivity. Clinical outcomes assessed included changes in insulin sensitivity, measured by the Matsuda Index, and oral glucose tolerance testing. The dietary intervention significantly altered bacterial taxa, with a reduction in pro-inflammatory genera and an increase in beneficial bacterial taxa. These shifts in the gut microbiome correlated with improved insulin sensitivity and glucose metabolism. Similarly, Valido et al,^34^ reported beneficial effects of probiotic supplementation, including reduced systemic inflammatory cytokines and improved gastrointestinal quality-of-life measures in 14 elite Swiss wheelchair athletes (11 with traumatic SCI). Clinical outcomes assessed included improvements in gastrointestinal symptoms (measured by the Gastrointestinal Quality of Life Index) and changes in inflammatory cytokine levels. On the molecular level, probiotic supplementation resulted in the reduction of 25/30 (83%) inflammatory markers and an increase in gut microbial alpha diversity. However, no major shifts in specific bacterial taxa were observed, indicating that changes in diversity and systemic inflammation were the dominant responses.

Using metabolomics, Yarar-Fisher et al,^35^ investigated the effects of a ketogenic diet, compared to a standard diet, in individuals with acute complete and incomplete SCI, observing increased anti-inflammatory serum metabolites and a decrease in fibrinogen levels, which correlated with enhanced motor function recovery, suggesting anti-inflammatory metabolic profiles may promote neurologic repair.

### Medications, hormones, and cell therapies

Five trials (22%) trials explored pharmacologic, hormonal, or cellular interventions, using proteomics, transcriptomics, metabolomics and metagenomics (table 3). These trials looked at the urobiome, sperm motility, neurologic recovery and circadian regulation.

**Table 3:** Summary of the findings for the medication, hormone, and cell therapy intervention studies included in the systematic review.

Author, Publication Year, Country, Type of Study	SCI Population, Age (SD)/(Range), Male (%)	Intervention	Sample, Organ-System, Omics Measured	Significant Findings, (↑) Upregulation, (↓) Downregulation	RoB Level
Martin-Rojas et al,^36^ 2020, Spain, RCT	46, 35, 38 (82.8)	Somatropin	Blood plasma proteins, inflammatory, proteomics	(↓) neurologic recovery associated proteins (APOA1, GC, ITIH4)	Moderate 2
Kostovski et al,^37^ 2018, Norway, RCT	6, 46 (27-60), 6 (100)	Melatonin	Peripheral blood mononuclear cells, circadian regulatory, transcriptomics	(↑) circadian rhythm regulators (PER1, PER2, REV-1α)	Moderate 2
Groah et al,^38^ 2023, United States, Pre-post study	23, NA, NA	LGG	Urine, Urinary, Metagenomics	(↓) pathogenic urobiome bacteria (*Escherichia-Shigella, Aerococcus*)	Moderate 4
Camargo et al,^39^ 2020, Brazil, Pre-post study	9, 40, 9 (100)	Probenecid	Semen, reproductive, proteomics	(↑) sperm motility enhancer (Cystatin-s) (↓) inflammatory marker (Biglycan)	Moderate 4
Singh et al,^40^ 2018, India, non-RCT	20 (acute SCI), NR, NR	Surgical fixation or stem cell adjuvant	Blood, neurologic and metabolic, metabolomics	(↑) energy and amino acid metabolism (acetone, acetate, succinate, lactate, isoleucine) (↓) recovery-inhibiting metabolite (glycine)	Moderate 3

*Abbreviations:* LGG = *Lactobacillus rhamnosus* GG; NA = not applicable; NR = not reported; RCT = randomized controlled trial; RoB = risk of bias; SCI = spinal cord injury.

Two RCTs assessed hormonal interventions: Martin-Rojas et al,^36^ reported growth hormone (GH) treatment (somatropin) significantly altered plasma protein profiles linked with improved motor scores (International Standards for Neurological Classification of Spinal Cord Injury Motor Index Score), demonstrating beneficial systemic proteomic responses as well as increases in plasma IGF-1 levels (GH treatment group). Proteomic analysis revealed differential expressed proteins involved in inflammation, homeostasis and coagulation pathways, with distinct biomarker signatures predictive of motor improvement (especially in GH treatment group). Kostovski et al,^37^ identified beneficial transcriptomic changes in circadian genes (PER1, PER2, BMAL1, and REV-ERBα) in peripheral blood mononuclear cells after melatonin administration, potentially ameliorating disrupted circadian rhythms common in tetraplegic patients. Clinical outcomes assessed included the normalization of sleep and shifting the peak gene expression closer to physiological nighttime patterns.

Groah et al^38^ utilized metagenomics to reveal beneficial shifts in the urinary microbiome after intravesical installation of *Lactobacillus rhamnosus GG* (LGG) in individuals with neurogenic bladder dysfunction. Changes in urinary symptoms (foul odor, cloudiness), measured by the Urinary Symptom Questionnaire for Neurogenic Bladder–Intermittent Catheterization (USQNB-IC), were assessed. On the molecular level, post-LGG treatment revealed a decrease in pathogenic genera (*Escherichia/Shigella*), alongside an increase in alpha diversity. In another urogenital pre-post study, Camargo et al^39^ demonstrated by proteomic analysis that oral probenecid treatment improved sperm motility (according to the World Health Organization 2010 criteria), associated with reduced seminal inflammation protein markers in male individuals with SCI. Singh et al^40^ identified specific serum metabolites linked with improved neurologic recovery (American Injury Association Impairment Scale grade) over 6 months after adjunctive stem cell therapy combined with surgical fixation, highlighting the value of metabolomics in predicting therapeutic responses. Of 28 analyzed metabolites, 7 (glycine, acetone, acetate, lactate, isoleucine, valine, and succinate) showed a significant correlation with neurologic recovery, particularly in the stem cell treatment group.

## Discussion

**Fig 2:**
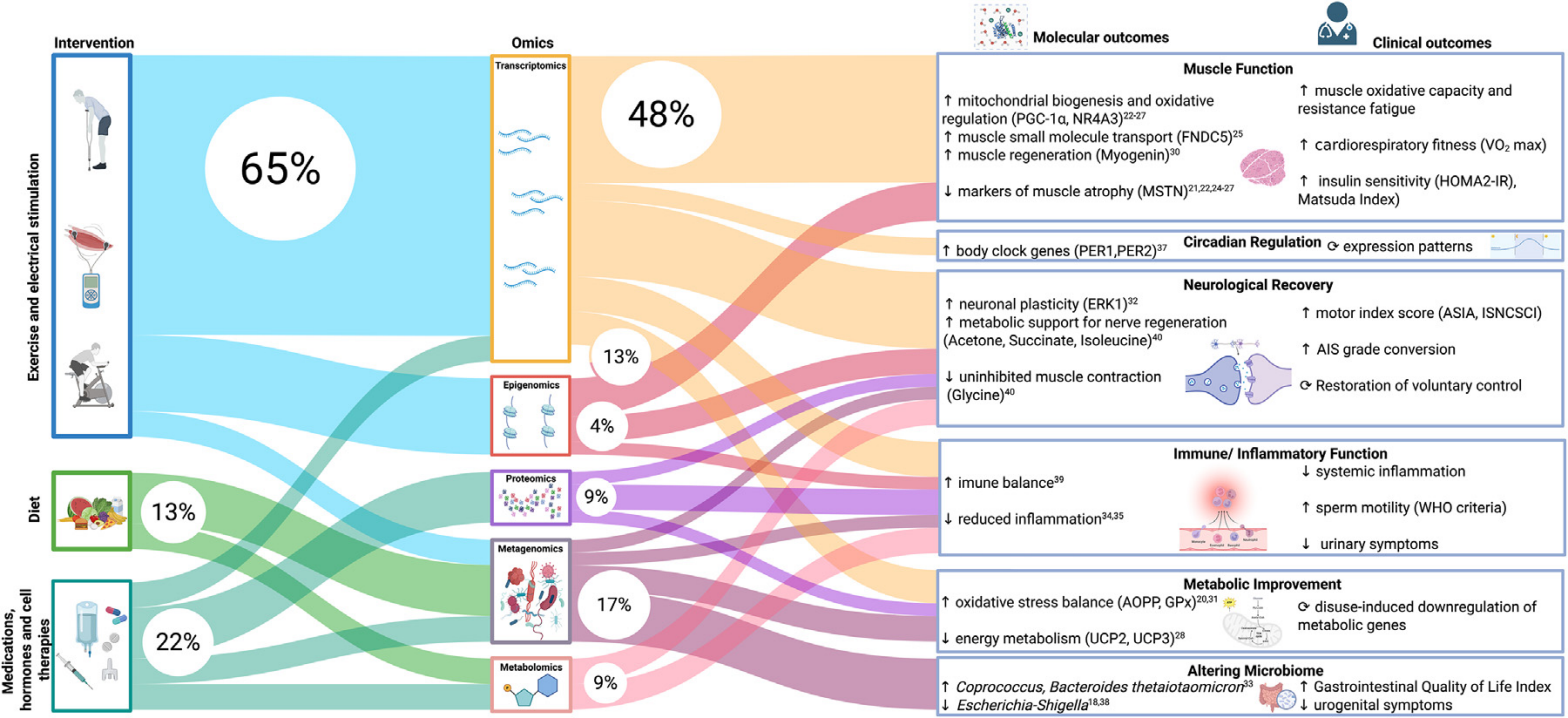
Graphical summary of potential omics-informed insights into rehabilitation interventions for spinal cord injury. This figure summarizes the distribution and impact of omics-informed rehabilitation interventions in individuals with spinal cord injury, based on a systematic review of 23 studies. Interventions are categorized into exercise and electrical stimulation (65%), dietary interventions (13%), and medications, hormones, and cell therapies (22%). These approaches were analyzed using diverse omics platforms: transcriptomics (48%), metagenomics (17%), proteomics (9%), metabolomics (9%), epigenomics (4%) and a combination of transcriptomics and epigenomics (13%). Key molecular and clinical outcomes affected by these interventions include improvements in muscle function, neurologic recovery, circadian regulation, immune/inflammatory function, metabolic processes, and microbiome composition. Created in BioRender. Stacul, A. (2025) https://BioRender.com/j4seg80.

This systematic review synthesizes current evidence regarding omics technologies—including transcriptomics, metagenomics, proteomics, metabolomics, and epigenomics—in monitoring molecular adaptations to rehabilitation interventions in individuals with SCI. By doing so, this review also aimed to examine the effectiveness of those interventions in promoting recovery, measured by omics analysis-derived molecular outcomes (graphical summary provided in figure 2). Most trials focused on exercise and electrical stimulation-based interventions that affected muscle activity, and a limited number of trials focused on dietary modification and the effects of medications, hormones, and cell therapies. The findings indicate that omics platforms are increasingly used to provide comprehensive molecular insights into rehabilitation efficacy, highlighting their potential role in developing personalized therapeutic strategies. Although the reviewed trials predominantly consisted of preliminary studies characterized by moderate methodological quality, significant molecular alterations linked to rehabilitation interventions were consistently reported, emphasizing omics’ sensitivity and utility in elucidating clinically relevant biological processes.

### Omics technologies in SCI rehabilitation research

Integration of omics technologies into SCI rehabilitation represents an innovative step toward precision medicine. Traditional rehabilitation approaches typically rely on clinical measures (eg, motor function scores, bowel management scales) that, while essential, may not adequately detect early molecular changes or subtle biological improvements after therapeutic interventions. However, most studies that used an omics approach have a retrospective nature and do not apply omics to prospectively monitor rehabilitation interventions, but rather re-analyze data from concluded studies. More recent applications demonstrate their underused potential for uncovering and mapping altered pathways, the discovery and identification of biomarkers, the observation of specific biomarkers, and giving mechanistic insights into rehabilitation interventions.^41^, ^42^, ^43^ This review highlights the growing number of applying omics platforms, with transcriptomics and metagenomics being the most frequently used (together 65%). Most studies employing omics technologies were transcriptomic analyses, predominantly used to characterize gene expression changes associated with skeletal muscle adaptations after exercise and electrical stimulation interventions.^19^^,^^21^, ^22^, ^23^, ^24^, ^25^, ^26^, ^27^, ^28^, ^29^, ^30^, ^31^, ^32^ Transcriptomics proved particularly valuable for monitoring molecular responses related to mitochondrial biogenesis, oxidative metabolism (eg, PGC-1α), and muscle atrophy reduction (eg, MSTN downregulation). These findings highlight transcriptomics’ potential not only as a biomarker discovery tool but also for validation of physiological adaptations.^44^ Metagenomics provided insights into microbiome alterations in response to exercise, dietary, and medication interventions, emphasizing the role of immune interactions with the urinary or gastrointestinal system.^18^^,^^33^^,^^34^^,^^38^ These microbiome shifts typically involved reductions in pro-inflammatory taxa (eg, *Escherichia-Shigella*) and increases in beneficial bacteria (eg, Bacteroides), correlating clinically with improved gastrointestinal function, metabolic control, and reduced systemic inflammation. Given the established importance of gut microbiota in regulating host immune and metabolic functions, these findings support the therapeutic potential of microbiome-targeted strategies in SCI rehabilitation.^45^ In contrast, proteomics and metabolomics analysis were less frequently employed (together 18%), but provided valuable complementary insights, identifying systemic biomarkers indicative of therapeutic efficacy, including improvements in neurorecovery, immune function, and reproductive health.^35^^,^^36^^,^^39^^,^^40^ Proteomics studies elucidated protein-level changes associated with hormonal and pharmacologic interventions, such as growth hormone treatment, enabling direct correlation of molecular profiles with motor improvement.^36^ Metabolomics similarly revealed metabolic biomarkers linked to improved neurologic outcomes after stem cell therapy, underscoring the translational potential of these systemic approaches for enhanced patient monitoring.^36^^,^^40^

Epigenomic analyses highlighted stable, long-term molecular adaptations to exercise and electrical stimulation interventions, capturing epigenetic modifications like DNA methylation, which potentially mediate sustained physiological benefits.^20^^,^^25^, ^26^, ^27^ Epigenomic profiling can thus complement transcriptomics by distinguishing immediate gene expression responses from more enduring molecular adaptations, potentially guiding long-term rehabilitation strategies tailored to individual epigenetic signatures.^46^, ^47^, ^48^, ^49^

### Effectiveness of rehabilitation interventions measured through omics

The reviewed trials predominantly investigated exercise-based and electrical stimulation interventions, effectively demonstrating their beneficial molecular impact on muscle and systemic metabolism. Due to the disuse of the musculoskeletal system after SCI, individuals having experienced such are at a greater risk of developing secondary complications such as diabetes, metabolic syndrome, or osteoporosis.^21^^,^^22^ Transcriptomic profiling consistently revealed increased expression of oxidative metabolic genes (eg, PGC-1α, NR4A3) alongside downregulation of muscle atrophy markers (MSTN), which correlated clinically with enhanced insulin sensitivity, aerobic capacity, and muscular endurance.^21^, ^22^, ^23^, ^24^, ^25^, ^26^, ^27^ Series of 5 studies^21^, ^22^, ^23^, ^24^, ^25^ on FES, demonstrates the potential of transcriptomics to monitor molecular changes in paralyzed muscle tissue. By monitoring early on the individual molecular changes, the time of beneficial targeted intervention for gene modulation can be applied to reduce the risk of conversion by adaptation of tissue and reduce the risk of metabolic disease.^50^ Furthermore, transcriptomic analysis after exercise and electrical stimulation interventions has emerged as a method for evaluating metabolic shifts in skeletal muscle. Electrical stimulation not only induces muscle contractions but also triggers a shift from glycolytic, fast-twitch fibers to more fatigue-resistant oxidative fibers, suggesting a shift in muscle fiber composition and metabolic function. While such changes can also be seen without the usage of transcriptomics, having the opportunity to analyze specific biomarkers can lead to earlier detection and target beneficial adaptations of shifts that may forecast later recovery. Furthermore, this could also lead to novel approaches in regenerative rehabilitation procedures. Severe osteoporosis in paralyzed muscles also leads to fractures occurring more often, even though small tasks, due to the loss of muscle mass.^21^^,^^22^^,^^51^^,^^52^ Tailoring intervention protocols specifically for the detected composition of muscle is crucial to not inflict bone damage. Collectively, these studies demonstrate that transcriptomic profiling can serve as a reliable indicator of how interventions affect muscle and related tissues. Additional transcriptomic studies regarding exercise and electrical stimulation-based intervention^28^, ^29^, ^30^, ^31^, ^32^ have explored a broader range of biological responses, including shifts in neuromuscular signaling, adipose tissue metabolism, and other aspects of systemic function.

In addition to the transcriptomic data analyses, epigenomic investigations provide further insights into both immediate and long-term molecular adaptations induced by rehabilitation.^20^^,^^26^^,^^27^ These approaches capture more stable modifications in gene regulation that reflect sustained benefits from interventions, such as improvements in muscle plasticity and overall metabolic balance. The integration of immediate responses detected through transcriptomics, with the enduring changes revealed by epigenomic analyses, could be helpful in clinical practice. Currently, sustainability cannot be measured clinically. Being able to detect it early on could be helpful for clinicians to develop comprehensive monitoring frameworks consisting of multiple layers.

In addition to these insights gained from muscle tissue, omics technologies can also provide valuable information regarding other aspects of molecular changes through intervention in SCI rehabilitation. In a previous systematic review, the gut and urinary tract microbiome profiles of individuals with SCI were different from those of uninjured individuals, and there was a higher abundance of inflammation-associated microorganisms in SCI.^53^ The use of metagenomics has been employed to monitor changes in the gut and urinary tract microbiome in response to exoskeleton-assisted walking, dietary interventions, and the intravesical installation of LGG.^18^^,^^33^^,^^34^^,^^38^ Studies have demonstrated that targeted nutritional strategies, such as a low-carbohydrate, high-protein diet or probiotic supplementation, can lead to an increase in beneficial microbial populations and a reduction in pro-inflammatory taxa.^33^^,^^34^ Similarly, exoskeleton-assisted walking has been linked to shifts in the gut microbiota, which is associated with improvements in bowel function and a modulation of systemic inflammation.^18^

Interventions involving medications,^37^^,^^39^ hormones,^36^ or cellular therapies^40^ provided compelling preliminary evidence that omics analyses could sensitively detect molecular changes predictive of functional recovery. For instance, proteomic and metabolomic studies identified biomarkers correlating directly with clinical improvement after growth hormone treatment and stem cell therapies, offering promising molecular predictors for therapeutic responsiveness. Systemic dysfunctions in the immune and endocrine systems are documented in SCI and manifest as chronic inflammation,^54^, ^55^, ^56^ immune cell dysfunction,^57^ increased risks of obesity,^55^ osteoporosis,^56^^,^^58^ and autonomic dysfunctions.^59^ Use of omics methods, as seen in this review, growth hormone treatments, and stem cell adjuvant therapies have been associated with distinct proteomic signatures and metabolite profiles that correlate with neurologic recovery. Such findings underline the substantial potential of omics in refining therapeutic protocols, allowing clinicians to anticipate patient-specific responses more accurately and to personalize interventions accordingly.

### Strengths and limitations

A primary strength of this review is its comprehensive synthesis across multiple omics platforms, highlighting distinct yet complementary contributions to understanding SCI rehabilitation mechanisms. This integrative perspective underscores “omics” potential in precision medicine, offering novel biomarkers and pathways for clinical exploitation. Additionally, the review provides critical methodological insights by identifying common strengths and limitations of current omics applications.

Despite the promising molecular insights presented, important limitations must be acknowledged. First, most included trials exhibited moderate methodological quality, predominantly due to incomplete blinding, limited randomization details, and inadequate justification of sample sizes, reducing confidence in attributing observed molecular changes solely to interventions. The NIH Quality Assessment Tools evaluated the overall rigor of clinical study designs but do not address omics-specific methodological quality. This represents a limitation of current evidence synthesis methods in omics-based rehabilitation research. Future work should develop validated quality criteria tailored to multi-omics studies. Furthermore, although quality assessment was performed independently by 2 reviewers, formal inter-rater agreement statistics were not calculated, which may limit quantitative evaluation of concordance between the reviewers. Second, heterogeneity in participant characteristics (injury completeness, duration, baseline health), intervention protocols, and omics methodologies limited direct comparisons between studies. Furthermore, most included studies were small-scale, short-term interventions, potentially restricting insights into long-term molecular adaptations. Standardized and harmonized “omics” sampling and analytical protocols were generally lacking, complicating cross-study synthesis and interpretation. Differences in platforms, databases, or bioinformatics tools can result in different interpretations of the same raw “omics” data.

Lastly, while omics platforms provide mechanistic resolution beyond traditional clinical metrics, their near-term clinical utility remains limited by cost, assay complexity (expertise), and turnaround times. Some rehabilitation outcomes, such as increases in strength, muscle mass, and functional independence, can already be measured reliably using standard clinical tools. At present, omics may be most valuable for identifying mechanistic responders, clarifying biological pathways, and generating precision-medicine hypotheses rather than guiding routine clinical decisions. However, the rapid miniaturization of sequencing and proteomic technologies, coupled with decreasing costs, is steadily narrowing the translational gap and may enable feasible clinical integration in the future.

### Future research directions

Future research should prioritize high-quality RCTs with rigorous methodological standards, clearly defined omics-driven endpoints, and standardized analytical procedures. Given the complexity and heterogeneity inherent to SCI rehabilitation, longitudinal cohort studies using large, well-characterized patient populations will be essential to capture comprehensive molecular profiles across the rehabilitation trajectory. Prospective longitudinal studies, such as those facilitated by established biobanks, are particularly valuable because they enable systematic, repeated sampling over extended periods, providing insights into the temporal dynamics of molecular changes and their sustained clinical relevance.

Moreover, leveraging routinely collected biospecimens from existing biobanks offers a robust platform for the detailed molecular characterization of SCI populations at scale. For instance, the Swiss Spinal Cord Injury Cohort Study and the Swiss Spinal Cord Injury Cohort Study Biobank, which systematically collects biological samples linked to clinical, functional, and demographic data, present an unparalleled opportunity for omics integration into routine clinical care and research.^60^ Utilization of biobanking samples allows researchers to perform multi-layered molecular analyses, integrating genomic, epigenomic, transcriptomic, proteomic, metabolomic, and metagenomic data, with high precision and reproducibility. Such an integrated multi-omics approach can significantly enhance the understanding of SCI pathophysiology, reveal novel biomarkers predictive of rehabilitation outcomes, and ultimately inform personalized intervention strategies.

Future studies should also emphasize methodological harmonization, particularly regarding biospecimen collection, processing, and omics data analysis, thereby facilitating cross-study comparisons and translational utility. Finally, investigating baseline omics signatures predictive of treatment responsiveness will enable precise patient stratification and the development of tailored rehabilitation programs, further advancing the promise of precision medicine in SCI rehabilitation.

## Conclusion

This systematic review highlights the potential of integrating omics technologies into rehabilitation interventions for SCI. By revealing clinically significant molecular adaptations, omics platforms, particularly transcriptomics and metagenomics, provide tools capable of elucidating underlying biological processes involved in rehabilitation-induced recovery. The molecular insights derived from these technologies will enhance our understanding of rehabilitation mechanisms and offer prospects for the development of individualized treatment strategies.

Although omics methodologies are not integrated into clinical rehabilitation practice, the preliminary evidence presented underscores their potential in shaping future rehabilitation strategies toward precision medicine. The systematic identification of molecular biomarkers and adaptive molecular signatures provides a critical foundation for targeted therapeutic approaches, enabling clinicians to customize rehabilitation programs based on patient-specific molecular profiles and responsiveness patterns.

Advancing the application of omics in clinical rehabilitation requires strategic investments in large-scale, longitudinal studies and biobank-supported research to validate biomarker panels comprehensively and reliably. Future research should prioritize methodological harmonization and the integration of multi-omics analyses to improve reproducibility, translational relevance, and patient-specific predictive capabilities.

Ultimately, the implementation of omics-driven personalized rehabilitation holds the promise of significantly enhancing functional recovery, improving quality of life, and reducing secondary complications for individuals living with spinal cord injury. Continued efforts in methodological refinement and collaborative integration of omics into clinical protocols represent a critical step toward achieving genuinely precision-driven, patient-centered rehabilitation outcomes.

## Appendix Supplementary materials

Image, application 1

Image, application 2

## Footnotes

Supplementary material associated with this article can be found, in the online version, at doi:10.1016/j.arrct.2026.100598.
