# Supplementary material for: Precision Rehabilitation in Spinal Cord Injury: A Systematic Review of Omics Applications for Intervention Monitoring in Spinal Cord Injury
Source: Arch Rehabil Res Clin Transl. 2026 Feb 16;8(2):100598. doi: 10.1016/j.arrct.2026.100598 (PMC13282838; doi:10.1016/j.arrct.2026.100598)
Supplement: Supplementary file 1 [file mmc1.docx]

**Supplemental appendix S1. Full Search Strategy per Database**

**Overview databases & results**

Date last searched **27.11.2024**

|  | Before deduplication | After deduplication |
| --- | --- | --- |
| Embase.com | 2041 | 1768 |
| Medline Ovid | 2053 | 1052 |
| Web of Science | 1927 | 1391 |
| Total | 6021 | 4211 |

**1810 duplicates**

**MEDLINE (Ovid) and EMBASE (OvidSP)**

exp Computational Biology/ OR exp Genome/ OR Gene Expression Regulation/ OR exp Transcriptome OR (((gene or genome or RNA or DNA or whole genome or metagenome or high-throughput) adj3 sequenc*) OR (gene adj3 (expression* or regulation* or profiling*)) OR epigenetic* or genomic* or microbio* or transcriptom* or proteom* or epigenom* or metabolom* or bioinformatic* or systems biology).ab,ti.

AND

exp Spinal Cord Injuries/ OR exp Paraplegia/ OR exp Quadriplegia/ OR (((spine or spinal) adj3 (injur* or trauma* or damag*)) or (spinal cord adj3 (contusion* or laceration* or transection* or lesion* or trauma*)) or ((spine or spinal or vertebra*) adj3 (fracture* or trauma* or injur* or damage*)) or paraplegi* or quadriplegi* or tetraplegi*).ab,ti.

NOT

(letter or news or comment or editorial or congres* or abstract*).pt. OR exp Animals/ or (rodent* or mice or mouse or rat* or murine or zebrafish or animal model*).ab,ti. not exp Humans/

**WEB OF SCIENCE**

TS=(((((spine or spinal) NEAR/3 (injur* or trauma* or damag*)) OR ("spinal cord" NEAR/3 (disease* or disorder* or contusion* or laceration* or transection* or lesion* or trauma* or post-trauma* or ischemi* or ischaemi*)) OR ((spine or spinal or vertebra*) NEAR/3 (fracture* or trauma* or injur* or damage* or wound*)) OR paraplegi* OR quadriplegi* OR tetraplegi* or wheelchair* or paralympi* OR para-athlet* OR parathlet* OR para-sport* )) AND (bioinformati* OR "gene mapping" OR "gene expression" OR transcriptom* OR proteom* OR metabolom* OR microbio* OR (sequenc* NEAR/3 (gene OR genome OR rna OR dna OR "whole genome" OR metagenome OR "high throughput")) OR (gene NEAR/3 (expression* OR regulation* OR profiling*)) OR epigenetic* OR genomic* OR "computational biology" OR "systems biology") NOT (animal* OR rodent* or mice or mouse or rat* or murine or zebrafish or animal model*))
