# Supplementary material for: Precision Rehabilitation in Spinal Cord Injury: A Systematic Review of Omics Applications for Intervention Monitoring in Spinal Cord Injury
Source: Arch Rehabil Res Clin Transl. 2026 Feb 16;8(2):100598. doi: 10.1016/j.arrct.2026.100598 (PMC13282838; doi:10.1016/j.arrct.2026.100598)
Supplement: Supplementary file 2 [file mmc2.docx]

**Supplemental Table 1. Risk of bias of interventional studies in the rehabilitation with use of omics technologies in individuals with spinal cord injury using the National Heart Lung and Blood Institute Quality Assessment Tool.**

| **Lead Author, Publication (Supplemental Reference); Year** | **Study Design** | **1** | **2** | **3** | **4** | **5** | **6** | **7** | **8** | **9** | **10** | **11** | **12** | **13** | **14** | **Risk of bias (Total score)^1^** | **Study**  **Level** |
| --- | --- | --- | --- | --- | --- | --- | --- | --- | --- | --- | --- | --- | --- | --- | --- | --- | --- |
| Valido (2024) | Pilot-RCT | Y | Y | N | N | N | Y | Y | Y | Y | Y | Y | N | Y | Y | Moderate (10/14; 71.42%) | 2 |
| Li (2022) | RCT | Y | Y | Y | Y | Y | N | Y | Y | Y | Y | Y | N | Y | Y | Low (12/14; 85.71%) | 1 |
| Goldhardt (2019) | RCT | N | N | N | N | Y | Y | Y | Y | Y | Y | Y | N | Y | NR | Moderate (8/14; 57.14%) | 2 |
| Kostovski (2018) | RCT | Y | N | N | Y | CD | Y | Y | Y | Y | Y | Y | N | Y | Y | Moderate (10/14; 71.42%) | 2 |
| Martin-Rojas (2020) | RCT | N | Y | Y | CD | CD | Y | Y | Y | Y | Y | Y | N | Y | NR | Moderate (9/14; 64.28%) | 2 |
| Nightingale (2017) | RCT | N | Y | N | N | N | Y | Y | Y | Y | Y | Y | N | Y | NR | Moderate (8/14; 57.14%) | 2 |
| Hu (2024) | RCT | N | Y | Y | N | Y | Y | Y | Y | Y | Y | Y | N | Y | N | Moderate (10/14; 71.42%) | 2 |
| Yarar-Fisher (2018) | Pilot-RCT | Y | N | N | N | N | Y | Y | Y | Y | Y | Y | N | Y | Y | Moderate (9/14; 64.28%) | 2 |
| Hjeltnes (1999) | Non-RCT | N | N | N | N | N | N | Y | Y | Y | Y | Y | CD | Y | Y | Moderate (7/14; 50%) | 3 |
| Lammers (2012) | Non-RCT | N | N | N | N | N | N | Y | Y | Y | Y | Y | CD | Y | Y | Moderate (7/14; 50%) | 3 |
| Singh (2018) | Non-RCT | N | CD | N | N | N | Y | Y | Y | Y | N | Y | Y | Y | Y | Moderate (8/14; 57.14%) | 3 |
| Petrie (2016) | Non-RCT | N | N | N | N | N | Y | Y | Y | Y | Y | Y | CD | Y | Y | Moderate (8/14; 57.14%) | 3 |
| Petrie (2014) | Non-RCT | N | N | N | N | N | N | Y | Y | Y | Y | Y | CD | Y | Y | Moderate (7/14; 50%) | 3 |
| Vissing (2005) | Pre-post | N | Y | Y | CD | N | Y | Y | N | Y | Y | Y | NA | - | - | Moderate (7/12; 58.34%) | 4 |
| Groah (2023) | Pre-post | Y | Y | Y | CD | N | Y | Y | N | Y | Y | N | NA | - | - | Moderate (7/12; 58.34%) | 4 |
| Petrie (2022) | Pre-post | Y | N | Y | Y | N | Y | Y | N | Y | Y | N | NA | - | - | Moderate (7/12; 58.34%) | 4 |
| Petrie (2024) | Pre-post | Y | Y | Y | Y | N | Y | Y | N | Y | Y | N | NA | - | - | Moderate (8/12; 66.67%) | 4 |
| Chang (2012) | Pre-post | Y | Y | Y | Y | N | Y | Y | N | Y | Y | N | NA | - | - | Moderate (8/12; 66.67%) | 4 |
| Camargo (2020) | Pre-post | Y | Y | Y | Y | N | Y | Y | N | Y | Y | N | NA | - | - | Moderate (8/12; 66.67%) | 4 |
| Ingles (2016) | Pre-post | Y | CD | Y | Y | N | Y | Y | Y | Y | Y | Y | NA | - | - | Moderate (9/12; 75%) | 4 |
| Petrie (2015) | Pre-post | Y | Y | Y | Y | N | Y | Y | N | Y | Y | N | NA | - | - | Moderate (8/12; 66.67%) | 4 |
| Petrie (2020) | Pre-post | Y | Y | Y | CD | N | Y | Y | N | Y | Y | Y | NA | - | - | Moderate (8/12; 66.67%) | 4 |
| Petrie (2014) | Pre-post | Y | N | Y | CD | N | Y | Y | N | Y | Y | N | NA | - | - | Moderate (6/12; 50%) | 4 |
| Abbreviations: CD, cannot determine; NA, not applicable; NR, not reported; RCT, randomised controlled trial; pRCT, pilot RCT  ^1^Risk of bias rating (Low (75-100%), Moderate (50-74%), or High (0-49%))  **Criteria used to assess risk of bias of controlled clinical trials:**  1. Was the study described as randomized, a randomized trial, a randomized clinical trial, or an RCT?  2. Was the method of randomization adequate (i.e., use of randomly generated assignment)?  3. Was the treatment allocation concealed (so that assignments could not be predicted)?  4. Were study participants and providers blinded to treatment group assignment?  5. Were the people assessing the outcomes blinded to the participants' group assignments?  6. Were the groups similar at baseline on important characteristics that could affect outcomes (e.g., demographics, risk factors, co-morbid conditions)?  7. Was the overall drop-out rate from the study at endpoint 20% or lower of the number allocated to treatment?  8. Was the differential drop-out rate (between treatment groups) at endpoint 15 percentage points or lower?  9. Was there high adherence to the intervention protocols for each treatment group?  10. Were other interventions avoided or similar in the groups (e.g., similar background treatments)?  11. Were outcomes assessed using valid and reliable measures, implemented consistently across all study participants?  12. Did the authors report that the sample size was sufficiently large to be able to detect a difference in the main outcome between groups with at least 80% power?  13. Were outcomes reported or subgroups analyzed prespecified (i.e., identified before analyses were conducted)?  14. Were all randomized participants analyzed in the group to which they were originally assigned, i.e., did they use an intention-to-treat analysis?  **Criteria used to assess risk of bias of pre-post study without control group:**  1. Was the study question or objective clearly stated?  2. Were eligibility/selection criteria for the study population prespecified and clearly described?  3. Were the participants in the study representative of those who would be eligible for the test/service/intervention in the general or clinical population of interest?  4. Were all eligible participants that met the prespecified entry criteria enrolled?  5. Was the sample size sufficiently large to provide confidence in the findings?  6. Was the test/service/intervention clearly described and delivered consistently across the study population?  7. Were the outcome measures prespecified, clearly defined, valid, reliable, and assessed consistently across all study participants?  8. Were the people assessing the outcomes blinded to the participants' exposures/interventions?  9. Was the loss to follow-up after baseline 20% or less? Were those lost to follow-up accounted for in the analysis?  10. Did the statistical methods examine changes in outcome measures from before to after the intervention? Were statistical tests done that provided p values for the pre-to-post changes?  11. Were outcome measures of interest taken multiple times before the intervention and multiple times after the intervention (i.e., did they use an interrupted time-series design)?  12. If the intervention was conducted at a group level (e.g., a whole hospital, a community, etc.) did the statistical analysis take into account the use of individual-level data to determine effects at the group level? | | | | | | | | | | | | | | | | | |

| **Supplemental Table 2.** **Eligible study designs and criteria for classifying the level of evidence for individual studies.** | | | |
| --- | --- | --- | --- |
| **Level of evidence** | **Study design** | **Study quality score based on NIH** | **Description study design** |
| Level 1 study | Randomized controlled trial | Low risk of bias | Using within-subjects comparison with randomized conditions or cross-over designs |
| Level 2 study | Randomized controlled trial | Moderate risk of bias | Using within-subjects comparison with randomized conditions or cross-over designs |
|  | Non-randomized controlled trial | Low risk of bias | Comparing intervention vs. control groups (not randomly allocated) |
|  | Prospective cohort study | Low risk of bias | Longitudinally comparing at least two similar groups (one exposed one unexposed) |
|  | Case-control study | Low risk of bias | Using a case-control study design to compare two types of diet/dietary patterns |
| Level 3 study | Randomized controlled trial | High risk of bias | Using within-subjects comparison with randomized conditions or cross-over designs |
|  | Non-randomized controlled trial | Moderate risk of bias | Comparing intervention vs. control groups (not randomly allocated) |
|  | Prospective cohort study | Moderate risk of bias | Longitudinally comparing at least two similar groups (one exposed one unexposed) |
|  | Retrospective cohort study | Low/moderate risk of bias | Retrospectively comparing an exposed/interventional group to a historical control group |
|  | Case-control study | Moderate risk of bias | Using a case-control study design to compare two types of diet/dietary patterns |
|  | Pre-post study | Low risk of bias | Using a baseline measure, intervention and a post-test in a single group |
| Level 4 study | Non-randomized controlled trial | High risk of bias | Comparing intervention vs. control groups (not randomly allocated) |
|  | Prospective cohort study | High risk of bias | Longitudinally comparing at least two similar groups (one exposed one unexposed) |
|  | Retrospective cohort study | High risk of bias | Retrospectively comparing an exposed/interventional group to a historical control group |
|  | Pre-post study | Moderate or high risk of bias | Using a baseline measure, intervention and a post-test in a single group |
|  | Cross-sectional study | Regardless of the quality score | Comparing two groups exposed and unexposed to specific diet/micro- or macronutrient |
